# Gunshot Wounds and Their Treatment

**Published:** 1897-10

**Authors:** H. R. Chislett


### Selections and Abstracts.

### GUNSHOT WOUNDS AND THEIR TREATMENT.

The most interesting feature of gunshot wounds is, that most freshly made ones are aseptic. As Esmarch says: "The damage done by a bullet is in its passage, the harm that is added comes mostly from the examiner's probe or fingers." The main object of the present paper is to emphasize these truths and to outline a plan of treatment which will tend to do away with injudicious manipulation of shot wounds. It seems a very difficult lesson to learn that in most cases a bullet is like any other aseptic foreign body and may remain buried in tissues almost indefinitely without special harm. Before taking up the treatment, however, or describing the conditions in which exploration is necessary, let us take a brief glance at the general characteristics of shot wounds, and the conditions which influence them. Our subject shall be limited to the wounds inflicted by small arms, they being the most common in civil practice.

While essentially a combination of punctured and lacerated wounds, shot wounds may have the characteristics of a simple contusion or of an incised wound. As met with in civil practice they vary from simple contusions to frightful lacerations, the character being influenced by the size, shape, and velocity of the missile, the distance from which the shot is fired, the location of the injury and the plane at which it strikes the body. The influence exerted by the size is too obvious to demand explanation. The form of the ball is of very great importance, and by its form we mean not only the original shape but the change which it may have undergone from its contact with intervening bodies. Most of the bullets of the present day are conical in shape, and the course being far more steady it is less frequently deviated than the old round balls. The pointed apex, the rotary motion, and the great velocity enables a conical ball to pierce tissues with more ease, and while the wound of entrance may appear smaller, the internal destruction is far greater than that inflicted by round balls. When lead balls have come in contact with hard substances, they are sometimes rendered peculiarly distorted and lacerate the tissues just in proportion to their irregularities. At close quarters and in direct injuries, the difference in the form of the ball makes less difference, the greatest danger being the entrance of clothing, wadding or powder. Speaking in a general way, the shorter the range the more pronounced the external laceration. The position occupied by the patient at the time of the reception of the injury should be considered in connection with the velocity of the ball and the distance at which the shot was fired, because, depending upon this position, a ball may travel through several parts of the body and thus give evidence that numerous shots have been fired. It must be borne in mind also in this connection that while a conical ball at full speed will destroy and pass through all human tissues, those of less speed (and round balls) may be deviated from their course by a projection of bone or a contracted tendon. These may likewise cause a splitting of the ball into fragments, in which case we may have but one wound of entrance and several of exit. Before describing the objects of attainment in an examination of shot wound, it may be well to describe a typical appearance of the wounds of entrance and of exit. The wound of entrance in a direct injury where the ball strikes squarely is usually round or stellate in form, about the size of or slightly smaller than the missile, the edges being darkened and inverted. The wound of exit is commonly larger than that of entrance, more irregular with everted edges and showing less contusion but more laceration. We have already seen the conditions which may entirely change these characteristics.

### DIAGNOSIS AND EXAMINATION.

The diagnosis of shot wounds is usually made for us, though there may be difficulty in determining whether one or more shots was fired. If we bear in mind, however, the general characteristics of the entrance and exit wounds and the conditions which modify them, it will not usually be difficult to determine this point. The examination should be conducted with the view of determining first, the presence of foreign bodies. If the wound of entrance is smaller or only slightly larger than the missile; if the edges are inverted and no more lacerated than the distance, the form, and size of the missile would seem to warrant; if the clothing over the seat of injury is perforated but not torn, and there are no shreds of clothing or wadding in sight, one would be pretty positive that no foreign body other than the ball had gained entrance. Second, the lodgment of the ball. This can as a rule be determined by the presence or absence of a wound of exit. Probing is entirely unnecessary. If there is more than one wound of exit one can not always be positive, for you can not know for certain into how many pieces the ball may have divided. Third, the organs and tissues involved in the injury. If you can determine the position of the patient at the the time of accident, the distance and direction from which the ball was fired, the force of the weapon and the nature of the ball, the probable course of the missile can be outlined. With this information, the sensations of the patient and the objective symptoms will enable one to decide with tolerable accuracy what tissues are injured. The extent of such injury can better be determined by the general symptoms.

SYMPTOMS AND PROGNOSIS.

The symptons to which the greatest attention should be paid, I mean the primary ones, are shock and hemorrhage. Shock is more grave and persistent when unattended by severe hemorrhage in wounds of the abdomen than in wounds of any other part of the body. Indeed, cases have been reported where slight contusions of the abdomen have produced profound and even fatal shock. The mental condition of the patient at the time of receiving the injury must largely influence this degree of shock, for I have seen cases with multiple perforations of the intestine retaining normal pulse and temperature, while others with simple flesh wounds have been in most profound collapse. The degree of subnormal temperature in these cases is of strong prognostic value. It is not infrequent to find the temperature 96 degrees F. or below. If it remain so for any length of time, no matter what the injury, the prognosis is grave. Fatal primary hemorrhage is not so frequent as one would suppose. The contusion, the torsion caused by the rotation of the ball, and syncope in severe cases are all good hemostatics and seem to control bleeding from vessels of ordinary size. Hemorrhage proves, of course, rapidly fatal when large vessels are severed, and should be regarded as one of the greatest dangers What may seem a slight flow during the period of shock may prove very serious during reaction. The secondary symptoms are those which follow the reaction. They include all conditions from simple surgical fever to the worst forms of infective surgical diseases and from simple traumatic inflammation to gangrene. We shall dismiss this part of our subject, as all of these infections should be treated on general principles, if they can not be prevented by perfect cleanliness at first examination. Speaking in a general way, the rate of mortality increases in proximity to the trunk. Injuries to the brain and spinal cord and perforations of abdominal and thoracic cavities, are always grave.

TREATMENT.

The undue importance which has been attached to the early removal of the missile by the laity and which the misdirected judgment of the majority of physicians still fosters, is the greatest stumbling-block to successful treatment. The simple presence of the ball is no indication for its removal, nor is it even an indication for probing.

I feel like once more emphasizing the great truth that the majority of freshly made shot wounds are aseptic to begin with. Do not, I beg of you, infect them by injudicious manipulation. Excluding the penetration of cavities, there are in reality but five conditions which render exploration, either with fingers or probes, justifiable. These are: First, a large wound which has been freely exposed to atmospheric influences; second, the presence of foreign bodies others than the ball; third, previous examinations without antiseptic precautions; fourth, extensive injury to large vessels, nerves or bone; fifth, infective inflammations already begun. In simple flesh wounds, either penetrating or perforating, when you see your patient soon after the accident, when no one else has yet examined him, when there is no marked hemorrhage or anesthesia present, thoroughly cleanse the surrounding parts the same as for any antiseptic operation, fill both wounds or all wounds carefully with iodoform or some dry dusting powder, apply a dry dressing and put the patient to bed, the injured part at absolute rest. It is well to bear in mind the possibility of infecting your wound during this cleansing process, so it is always best before beginning the scrubbing either to fill the wounds up with a dry powder or to gently pack them with a carbolized or mercuric gauze. This can be removed after the preparation and the extra precaution of a cleansing with hydrozone taken.

If, however, your patient was shot several hours before, if the wound is large and markedly lacerated, if the clothing at the site of the injury was torn and small shreds could be detected in the wound, if the attending physician has been poking around for the ball for the last few hours, or if you have reason to suspect an injury to large vessels and nerves, give your patient an anesthetic, prepare the part for an antiseptic operation and if the wound be in an extremity, apply a constrictor above the injury. Follow that bullet tract with a probe and scalpel, making your incision sufficiently large to see what you are about. The finger, if surgically clean, is the best probe always. Remove all particles of dirt, clothing, wadding, or other foreign bodies, irrigating meanwhile either with a mild antiseptic solution or a sterilized normal salt solution. Examine the main blood vessels carefully, and if the artery is wounded ligate above and below and cut the vessel between the ligatures. If the vein is injured you must use your judgment in the individual case whether lateral ligature or suture would be preferable to double ligation and section. Stitch together the ends of injured nerves after careful removal of any hopelessly injured portions. After being sure of the thorough removal of all foreign matter including tissues traumatized beyond hope of recovery, the parts should be thoroughly cleansed with hydrozone and later irrigated with an antiseptic solution, preferably a 10 per cent solution of iodine.

The question of sutures is one of very great importance. My own practice is that if traumatism is very great, sutures should never be used. When there is a prospect of perfect regeneration of tissues which are only slightly traumatized, I introduce sutures but do not tie them, but pack the wound carefully with iodoform gauze for twenty-four or forty-eight hours to give the tissues every advantage without the tension of sutures. If upon second dressing the tissues look healthy, the sutures are tightened, thus getting union by the method of secondary suture.

Should suppuration occur either before or after secondary suture, the only treatment is the establishment of perfect drainage. My experience has been that this care to avoid tension of damaged tissues gives us the least likelihood to suppurative inflammation. Badly traumatized tissues united by primary suture will, in the majority of cases, suppurate even after the most stringent measures of surgical cleanliness.

Injuries of bone vary from simple fractures to the worst cases of compound comminuted fractures. Simple fractures should be treated on general principles. Compound fractures not markedly comminuted should be treated on general principles outlined in the treatment of the wounds of soft parts in addition to carefully splinting. When the bone is badly comminuted, operation is demanded. To avoid repetition I shall simply enumerate the main objects of attainment in the treatment. First, thorough antiseptic cleanliness; second, the removal of foreign bodies and detached splinters of bone; third, perfect remolding of fragments after removing sharp projections and irregularities with bone forceps, holding the opposing surfaces in contact with pins or suture; fourth, establishing adequate drainage, usually by leaving the wound open (this will, of course, depend upon the extent of the traumatism of the soft parts); fifth, an abundant antiseptic and absorbent dressing; sixth, the careful application of splints supported by plaster of Paris to maintain apposition and immobilization. Extensive comminution of bone when complicated with serious injury to blood vessels or nerves of an extremity, will of course demand amputation. Other conditions which may demand amputation are gangrene, whether simple from cutting off of the blood supply, or the acute, traumatic, spreading gangrene; acute infectious osteomyelitis developing from the points of comminution. In amputating after a shot injury, the same precaution is necessary as after machinery accidents, namely, remember the tissues are damaged far beyond what at first sight seems to be the limitation, so amputate sufficiently high to be sure that your flaps are in healthy, unhurt tissues.—H. R. CHISLETT, M. D., in *The Clinique*.

